# Genetic and functional characterization of the natural transformation system in Streptococcus constellatus

**DOI:** 10.1099/mic.0.001726

**Published:** 2026-06-18

**Authors:** Andreas Solberg Sagen, Kazi Shefaul Mulk Shawrob, Gabriela Salvadori, Roger Junges

**Affiliations:** 1 Institute of Oral Biology, Faculty of Dentistry, University of Oslo, Oslo, Norway; 2 Department of Public Health and Sport Sciences, Faculty of Social and Health Sciences, University of Inland Norway (INN), Elverum, Norway

**Keywords:** DNA transformation competence, gene editing, gene expression regulation, bacterial, quorum sensing, *Streptococcus*, *Streptococcus constellatus*

## Abstract

*Streptococcus constellatus* is an opportunistic pathogen frequently associated with abscess formation in various body sites. While the species has been shown to acquire exogenous DNA through natural transformation, functional analyses of its underlying mechanisms and optimized genetic editing protocols remain limited. Thus, our aim was to characterize the natural transformation system in *S. constellatus* and investigate environmental factors coordinating its activation. In addition, we sought to develop an optimized protocol for genome editing. Genomic analysis revealed that 73% of analyzed strains possess orthologs for essential competence regulon genes, with 58% harboring both a complete ComCDE-based operon and the putative transformation machinery required for natural competence. While all complete genomes harbored three copies of the master regulator *sigX*, the accessory regulator *comW* was seemingly absent. Lacking the peptide exporter *comAB*, we demonstrated that *S. constellatus* utilizes the bacteriocin transporter *silED* for competence-stimulating peptide export. Gene expression assays indicated system activation at peptide concentrations as low as 4 nM, with peak *sigX* expression obtained over 60 nM. With the goal of optimizing gene editing strategies, we developed a protocol utilizing rich media supplemented with BSA and calcium chloride, significantly increasing transformation frequencies. Furthermore, we observed that environmental stressors can upregulate the system, including hydrogen peroxide and subinhibitory concentrations of the antibiotics erythromycin, chloramphenicol and ampicillin. Given the increasing clinical relevance of the anginosus group, elucidating horizontal gene transfer mechanisms can provide critical insights into the evolutionary process and pathogenic potential of these species.

## Introduction

*Streptococcus anginosus* group (SAG) comprises three different species of streptococci: *Streptococcus anginosus*, *Streptococcus intermedius* and *Streptococcus constellatus*. Anginosus group species most notably colonize the oral cavity, throat, gastrointestinal and genital tracts as a commensal part of the human microbiome but can act as opportunistic pathogens causing non-invasive and invasive infections including pharyngitis, bacteraemia and abscesses in various body sites [1]. The ability of anginosus group species to thrive in low-oxygen and acidic environments has been proposed as an important adaptation that supports abscess formation, and they also present diverse defence mechanisms that can suppress the immune response [2,3]. Additionally, a large fraction of anginosus group genomes harbor a variety of mobile genetic elements and prophages, likely contributing to rapid adaptability to external stimuli on a population level [4,6]. In this context, the anginosus group, as a consequence of their plasticity and wide range of habitats in the human body, possesses a distinctive opportunity for horizontal gene transfer (HGT) across human microbiomes as they can interact with many species including priority human pathogens.

Anginosus species have been shown to naturally transform by incorporating exogenous DNA from the environment [7]. The natural transformation process and machinery relies on the ComDE two-component regulatory system in the mitis and anginosus group of streptococci, having most notably been studied in *Streptococcus pneumoniae* [8]. This system is based on a phosphorylase mechanism that is activated by the competence-stimulating peptide (CSP), whose precursor is encoded by the gene *comC*, when binding extracellularly to the histidine kinase ComD. Upon interaction with the peptide, a phosphorylation cascade activates the response regulator ComE [9]. Once activated, ComE controls the expression of early competence genes including the alternative sigma factor SigX, which then enables the transcription of the downstream cascade involved in DNA uptake, recombination, DNA repair, stress-response, and fratricide [8]. Activation of early genes includes *comW*, which plays a role in stabilizing SigX for late gene activation in *S. pneumoniae* [10]. Competence is also linked to predation via bacteriocin production by the BlpRH operon in *S. pneumoniae*, coordinated by the BlpC peptide [11], and by the lysis of neighboring non-competent cells through the expression of specialized cell wall hydrolases [12]. These hydrolases are co-expressed with immunity factors that protect competent cells from autolysis, thereby ensuring selective targeting. The resulting release of extracellular DNA generates a local pool of genetic material that can be internalized and integrated via homologous recombination, functionally coupling cell lysis to HGT [13]. In *S. pneumoniae*, the competence system has been shown to affect gene expression of as much as 4% of the genome [14,16]. For the anginosus group, reports are more limited; however, previous studies in *S. anginosus* showed that strain SK52 contains most of the known essential competence genes and is capable of transforming in laboratory conditions [17]. In *S. constellatus*, an ortholog of *comCDE* and three copies of *sigX* have been identified previously [7,15]. In addition, a recent study corroborated the functional CSP sequence in the *S. constellatus* type strain as a 16-amino acid peptide [18]. Analogous to the BlpRH operon in the pneumococcus, SAG species possess the *Streptococcus* Invasion Locus (Sil), under coordination of the SilCR peptide [19,20].

As interest in the anginosus group rapidly increases due to evidence of its virulence, such as abscess formation and potential tumor involvement [1,21], an expansion of the repertoire of genetic tools is likely to benefit the field. In addition, investigating HGT mechanisms in this group of species can provide novel insights into genome plasticity and survivability during the transition from commensal to pathogen. Here, we show that the majority of *S. constellatus* genomes present a complete competence regulon including the genes necessary for recombination. We further identified three copies of *sigX* in all complete genomes, with no *comW* present. Export of CSP relies on *silED* in this species, as the only transporter available for both CSP and SilCR, and we observed that *sigX* expression in response to synthetic CSP (sCSP) takes place already at low concentrations. Further, we present an optimized protocol for transformation of *S. constellatus* utilizing BSA and calcium chloride that significantly improves transformation frequencies for both plasmid and amplicons used as donor DNA. Finally, through the assessment of a panel of environmental stressors, we observed that hydrogen peroxide (H_2_O_2_) and subinhibitory concentrations of the antibiotics erythromycin, chloramphenicol, and ampicillin stimulate *sigX* expression, which may have important implications for habitat adaptation in this species coupled with repercussions related to antibiotic selection for treatment of infections.

## Methods

### Bacterial strains and culturing conditions

Bacterial strains, peptides and antibiotics utilized in the study are listed in Table S1, available in the online Supplementary Material. Peptides and antibiotics were dissolved and stored according to the instructions of the manufacturer. *Streptococcus constellatus* subsp. *constellatus* type strain CCUG 24889 and isogenic strains were generally cultured in tryptic soy broth (TSB; Oxoid, Basingstoke, UK) at 37 °C in a 5% CO_2_ humidified atmosphere. Brain-Heart Infusion (BHI; Oxoid, Basingstoke, UK) was prepared as described by the manufacturer, whereas THY was prepared from Todd–Hewitt broth (Oxoid, Basingstoke, UK) mixed with 0.2% yeast extract (Oxoid, Basingstoke, UK). Chemically defined medium (CDM) and C+Y-medium with yeast extract and BSA (C+Y_YB_) were prepared in accordance with the procedures described by Chang *et al*. [22] and Stevens *et al*. [23]. Incubation of petri dishes was performed in anaerobic conditions, utilizing the Anoxomat III jar system (Advanced Instruments LLC, Norwood, MA, USA), for 48 h as we observed more robust colonies, facilitating CFU determination. As described previously [24], bacterial pre-cultures for transformation and reporter assays were prepared by growing *S. constellatus* to optical density 600 (OD_600_)≈0.4 from fresh colonies, supplemented with 15% sterile glycerol and stored at −20 °C. When applicable, *S. anginosus* and *S. intermedius* strains were cultured using similar conditions.

### Reporter and mutant constructions

Mutants were constructed using overlapping PCR mutagenesis with sCSP as described previously by Junges *et al*. [24]. Oligonucleotides and donor DNA are listed in Table S2. Briefly, the promoter region upstream of *sigX* (SCSC_RS00080 - P*_sigX_*) was amplified and fused with a firefly luciferase gene (*fluc*) and *aad9* (spectinomycin resistance cassette), originated from pFW5-luc [25], and inserted upstream of the P*_sigX_* locus in an amplicon. Subsequently, the amplicon was ligated via overlapping PCR with two 2-kb flanking regions P*_sigX_* to generate an amplicon for transformation into the chromosome of CCUG 24889, thus inserting the reporter locus upstream of the intact *sigX* locus. Deletion mutants were constructed similarly by amplifying and fusing 2–3 kbps flanking regions with an *erm(B*) resistance cassette in place of the deletion locus with overlapping PCR. The final amplicon was then transformed into either CCUG 24889 or isogenic strains. Colonies for all mutants were then recovered in the appropriate selective antibiotic, and further genotype confirmation was performed with PCR. DNA and plasmid extractions were performed with DNeasy Blood and Tissue kit (Qiagen, Hilden, Germany) and the ZymoPure Plasmid Miniprep Kit (Zymo Research, Irvine, CA, USA), respectively, at times with an additional lysis step involving 30-min enzymatic treatment with 10 mg ml^−1^ lysozyme and 0.1 U ml^−1^ mutanolysin to maximize yield. Fragment purification was conducted with MinElute PCR Purification Kit (Qiagen, Hilden, Germany).

### Transformation and reporter assays

Initial protocols were adapted from previous reports in other streptococci [17,26]. Transformation assays were performed by growing a 1 : 100 dilution of preculture to an early log-phase (OD_600_≈0.04), then donor DNA and 250 nM sCSP were added in 200 µl total volume. Cultures were generally incubated for 2 h, then plated onto TSB agar with selective antibiotics and incubated for 48–72 h in anaerobic conditions. Transformation frequency was calculated as the ratio of CFU on selective agarose to CFU on non-selective agarose. Different conditions and parameters for transformation were assessed as described in the results section. As donor DNA, amplicon aSC011.3 (500 ng ml^−1^) and plasmid pRJ11 (5 µg ml^−1^), both conferring kanamycin resistance, were utilized generally. The aSC011.3 is a 3,409 bp amplicon with the kanamycin resistance gene *aphA-3* in the intergenic region between genes SCSC_RS06260 and SCSC_RS06255, whereas the plasmid pRJ11 is a 7,646 bp non-integrative plasmid containing the *aphA-3* kanamycin resistance gene as well. A second amplicon, aSC011.7, was also designed to integrate in the same intergenic region but contains a longer homologous flanking region making this amplicon a total of 7,097 bp. For general transformation, the 3,409 bp amplicon was utilized, unless otherwise stated. For reporter assays, pre-cultures of the reporters at OD_600_≈0.4 were diluted 1 : 100 in TSB with 100 nM d-luciferin (Synchem, Felsberg-Altenberg, Germany) in the presence or absence of different concentrations of sCSP (CSP A: DSRIRMGFDFSKLFGK) at varying cell densities and antibiotic concentrations. Aliquots of 200 µl were distributed in 96-well microtiter plates. The cultures were monitored in a plate reader (Synergy HT; BioTek, Winooski, VT) for 24 h at 37 °C at high luminescence sensitivity. In post-run analysis, the background values were subtracted, and luminescence (in relative light units -RLU) was adjusted to the OD_600_ value recorded.

### Growth under stress conditions

Antibiotic stress assays were performed in 96-well microtiter plates incubated in a plate reader with twofold serial dilutions of spectinomycin, kanamycin, erythromycin, tetracycline, vancomycin, ciprofloxacin, chlorhexidine, rifampicin, chloramphenicol, novobiocin or streptomycin. Initial concentrations are listed in Table S1. Similarly, the H_2_O_2_ experiment was performed using concentrations of 0.5, 1, 2, 3, 4 and 5 mM with dilutions freshly prepared from 3% concentrated H_2_O_2_. For testing the effects of pH, TSB solutions were adjusted prior to sterilization using HCl and NaOH, and recalibrated under sterile conditions after equilibration at 37 °C. All experiments included a control without treatment in addition to a blank medium control. Bacterial cultures were prepared from pre-cultures 1 : 100 and loaded onto each test group. Transformation assays were performed concomitantly in similar conditions using cultures with an OD_600_≈0.04 as described above. The results presented are representative of at least three independent biological experiments.

### RNA extraction, cDNA synthesis and RT-qPCR

Cells were harvested and RNA was extracted with a High-Pure RNA isolation kit (Roche) with an additional lysis step containing 10 mg ml^−1^ lysozyme, 100 U ml^−1^ mutanolysin and 10 mM Tris-HCl to maximize yield. Further, cDNA was synthesized using a First Strand cDNA synthesis kit (Thermo Fisher Scientific) following the manufacturer’s protocol with 1 µg RNA per reaction and an additional DNase treatment step. Gene expression was quantified using reverse transcript quantitative real-time polymerase chain reaction (RT-qPCR) with Maxima SYBR Green/ROX (Thermo Fisher Scientific) following manufacturer’s guidelines with 500 nM oligonucleotides, 10 ng cDNA and a three-step cycling protocol on an AriaMX Real-Time PCR system (Agilent, Santa Clara, USA). The primers were designed using the National Center for Biotechnology Information (NCBI) Primer-blast function with the relevant genes as template [27]. The quantification cycle (Cq) was normalized to the expression of a reference gene *gyrA* using Livak’s method [28].

### *In silico* regulon detection, bioinformatics and statistical analysis

Genomes of *S. constellatus* were obtained from the reference sequence database at NCBI in October 2024 and are listed in Table S3 [29,30]. Gene orthologs of the competence regulon were identified using a combination of blastn and tblastn using gene queries listed in Table S4 [31]. The resulting hits were manually curated with particular focus to ensure inclusion of less conserved and smaller genes such as *silCR* and *comC* and remove duplicates from closely related genes such as *comE* and *silB* [32]. The 100 bp of the upstream region of genes in *S. constellatus* CCUG 24889 known to be regulated by SigX in *S. pneumoniae* were extracted and analyzed using Multiple EM for Motif Elicitation (MEME) v5.5, followed by EMBOSS fuzznuc to refine a more specific search pattern using previously known cinbox motifs (for competence inducing) [16,33, 34]. Guided by qPCR data and an assembly of high-confidence motif binding sites, Find Individual Motif Occurrences (FIMO) was used to scan all the 33 genomes for high-confidence binding sites within 200 bp of a gene start codon [35]. The phylogenetic relationship between the strains of *S. constellatus* was determined using PHANTASM v1.1.3 with *S. pneumoniae* D39 as the outgroup [36]. Statistics and data visualization were either performed using R v4.4 or GraphPad Prism v10.2. For statistical analysis, the differences in treatment responses were analyzed using one-way repeated measures ANOVA or paired t-test across the changes in conditions. Data shown are representative of at least three independent biological experiments, each with at least three technical replicates, with details for the specific tests and outcomes described in each figure. Significance level was set at 0.05.

## Results

### The competence regulon in *S. constellatus*

**Fig. 1.**
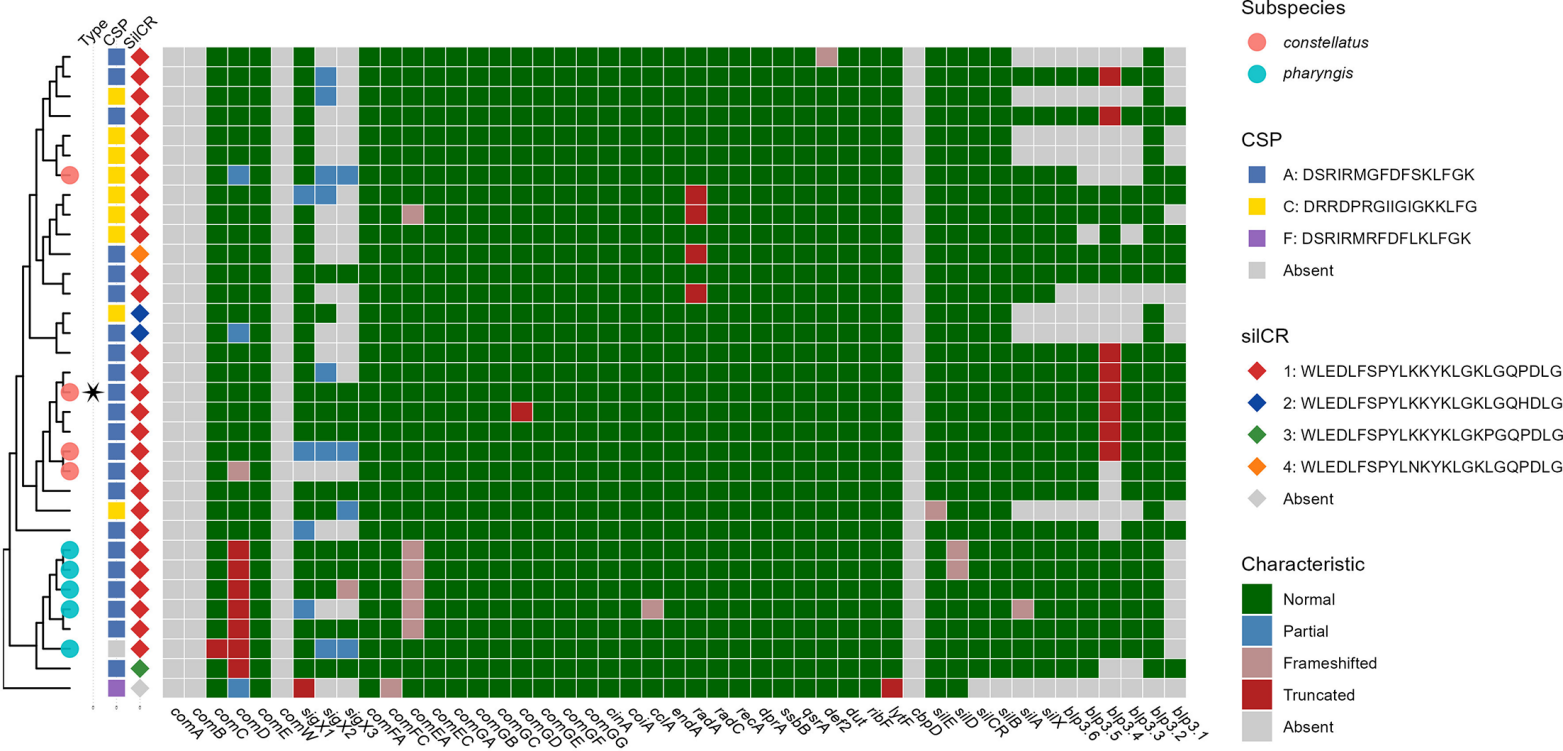
Distribution of competence-related genetic machinery in *S. constellatus*. The heatmap illustrates the status of genes involved in competence and fratricide systems. Gene characteristics are categorized as normal (green), partial (blue), frameshifted (brown), truncated (red) or absent (grey). These features are inferred from genomic data: partial denotes genes that are incompletely sequenced or assembled, lacking verified 5′ or 3′ termini; frameshifted indicates a reading frame disruption caused by an insertion or deletion and truncated signifies a gene which results in a shorter version of the protein being produced. Phylogenetic analysis based on core genes of *S. constellatus* strains was utilized to order the strains, whereas subspecies annotations derived from NCBI and CSP/SilCR isoforms have been assigned to each strain. The type strain CCUG 24889 is indicated by a star between the phylogenetic tree and the CSP classification.

The anginosus and the mitis group of streptococci present competence activation and bacteriocin production under regulation of the ComCDE and BlpRH operons, respectively [7,32]. In SAG, there is an analogous system of *blpA/B/C/H/R*, termed the Sil, coordinated by *silE/D/CR/B/A* [37]. The systems are structurally similar and likely have a similar function [20]. Initially, we mapped the competence regulon in 33 genomes of *S. constellatus* deposited in the databases of the NCBI, utilizing the known regulon of *S. pneumoniae* and the reported genes from *S. anginosus* as references [16,17]. Findings in *S. constellatus*, including a classification of the pheromones, identification of atypical features and missing genetic elements clustered by genotypic similarity of the strains, are summarized in Fig. 1. Only one peptide cleavage/export ATP synthase (ATP)-binding cassette transporters (ABC) transporter system was identified across all the analyzed genomes. The locus was identified upstream of a gene cluster coding for bacteriocins and immunity proteins, a histidine kinase and a transcription factor, which resembles the BlpAB cluster from *S. pneumoniae*. Together with the closer sequence similarity with BlpAB from *S. pneumoniae* and SilED from *S. anginosus* (Table S5), we putatively classified these genes as *silED* for *S. constellatus*.

Many strains of *S. constellatus* subsp. *pharyngis* presented a truncated *comD* and mutations leading to a frameshifted *comEA*. The truncation in *comD* takes place at the 3′ end of the gene, and a previous report shows that SigX can be activated despite the truncation. This is indicative that the lack of transformation observed for these strains is likely due to the frameshift in *comEA* [38]. It has previously been reported that species of the anginosus group have three copies of the *sigX* gene [15]. We found that 85% of the analyzed strains have at least one complete copy of *sigX* present. All completely assembled genomes analyzed in this study carried three copies of the *sigX* gene, whereas the other strains that appeared to lack three copies of *sigX* were draft assemblies. Thus, it is possible that the other copies are also present and functional but have not been reflected in the current sequencing data. If so, this is likely because of poor genome assembly due to the locus consisting of long repeating regions, particularly the 16S rRNA in the vicinity. In one of the genomes, *S. constellatus* subsp. *constellatus* SK53, no copy of *sigX* was identified, and one isolate *S. constellatus* SS_Bg39 presented only one partial copy of *sigX* at the edge of the contig. Further, the *sigX*-stabilizer *comW* was absent across the *S. constellatus* strains analyzed.

Three forms of putative mature CSPs were identified based on *comC* sequences, two previously identified – DSRIRMGFDFSKLFGK (CSP A) and DRRDPRGIIGIGKKLFG (CSP C) – with the first sequence being more common and also found in the type strain. A third not yet characterized sequence (DSRIRMRFDFLKLFGK), which we named CSP F following previous work by Lacroix [38], was found in *S. constellatus* SS_Bg39, which is likely an isoform originating from CSP A with two residue substitutions [38]. *S. anginosus* does not present the murein hydrolase CbpD that is well characterized in *S. pneumoniae*, but rather presents another peptidoglycan hydrolase named LytF, which has been suggested to be involved in fratricide in *Streptococcus gordonii* and *Streptococcus mutans* [13,39]. In all genomes surveyed of *S. constellatus*, an intact copy of *lytF* was identified, but not *cbpD* (Fig. 1). In summary, we have identified that 73% of the strains analyzed contain at least one normal copy of *silED*, *comCDE* and *sigX,* essential for competence development, and 58% of strains containing a seemingly complete regulon for transformation including *silED*, *comCDE*, *sigX*, *comFA/C*, *comEA/C*, *comG*, *cinA*, *coiA*, *cclA*, *endA*, *radA*, *dprA* and *ssbB*.

### Identification of SigX binding motifs and putative regulon

**Fig. 2.**
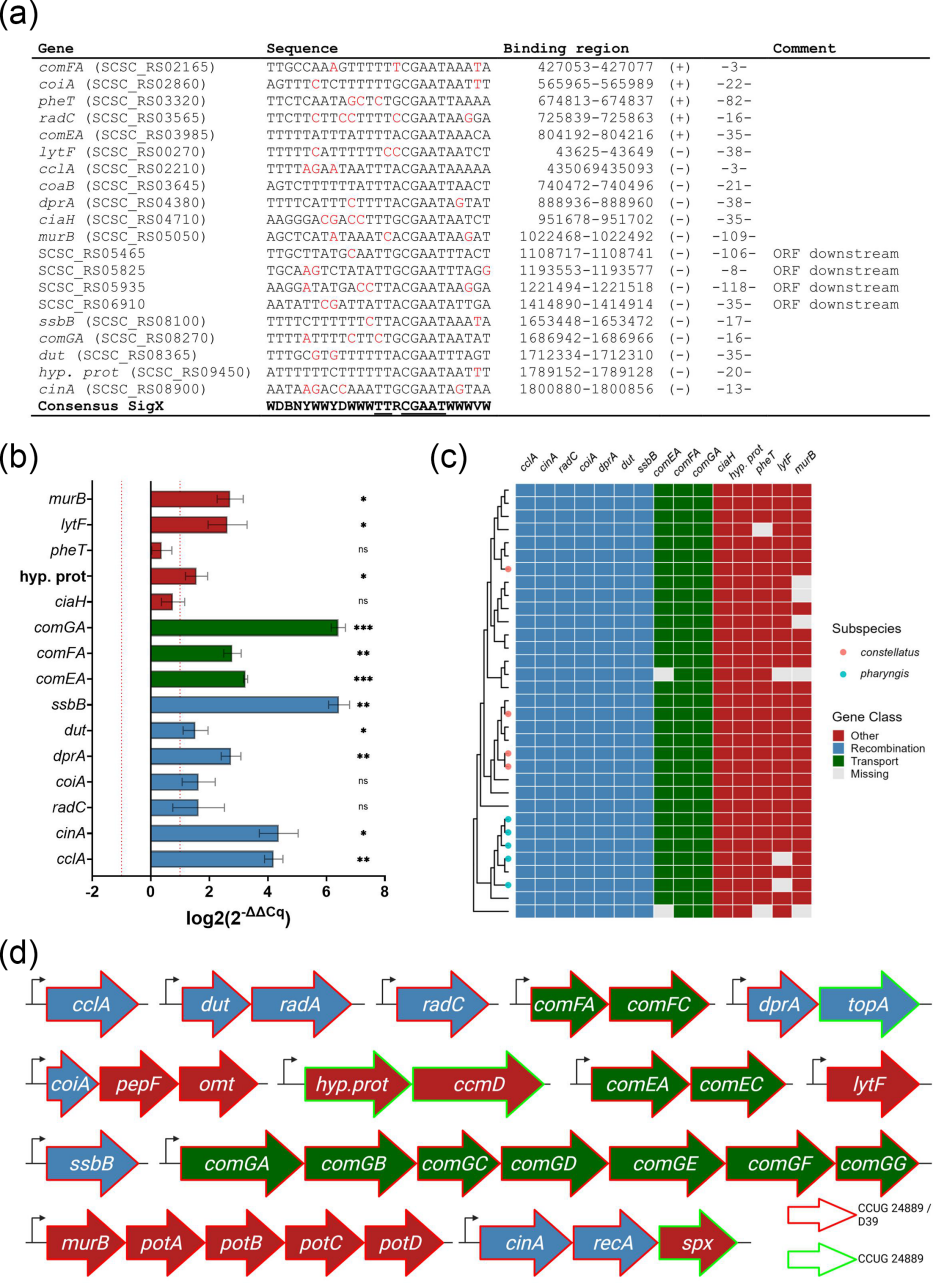
Characterization of the late competence regulon. (**a**) Alignment of the cinbox motif and IUPAC consensus sequence upstream of predicted late genes. (**b**) RT-qPCR analysis of CSP-induced late gene expression represented by Livak method (2^-ΔΔCq^). Data represent mean±sem for three biological replicates normalized to *gyrA*. Statistical significance tested between ΔCq of CSP-treated group with the ΔCq of the non-treated group using paired t-test (ns: not statistically significant, *P<0.05, **P<0.01, ***P<0.001). (**c**) Analysis of cinbox motifs identified across 33 genomes, categorized by functional class. (**d**) Genomic organization of gene clusters located downstream of identified cinboxes. Red outline indicates similar organization in type strain CCUG 24889 and *S. pneumoniae* D39, whereas green outline indicates unique organization in *S. constellatus*.

After binding of sufficient levels of CSP, a phosphorylation cascade activates expression of *sigX*, which further activates late genes related to DNA incorporation, recombination and fratricide [8]. Based on previous reports, a total of nine candidate genes were selected as part of the high-confidence late competence regulon: *cclA*, *coiA*, *cinA*, *comEA*, *comFA*, *comGA*, *dprA*, *dut* and *ssbB* [16]. The 100 bp upstream regions of these genes were extracted and analyzed using MEME to identify conserved motifs. By doing this, we identified a conserved 10 bp motif**,** TTTRCGAATA. As expected, this sequence motif is similar to the 8 bp sequence, TACGAATA, consensus sequence found in *S. pneumoniae*, *Streptococcus mitis*, *Streptococcus pyogenes* and *Streptococcus infantarius* [16,40]. Additionally, we observed a thymine-rich upstream and adenine-rich downstream regions in line with the extended cinbox reported by Slager *et al*. [16]. Utilizing the promoter sequences of the high-confidence SigX regulon described above, we refined an extended cinbox motif for *S. constellatus* – WDBNNNHNNNHHYYNCGAATWDDND – and performed searches in the entire genome of the type strain. We combined results and identified several motifs upstream of genes or ORFs within 100 bp, and some beyond, including *pheT*, *lytF*, *coaB*, *ciaH*, *murB* and hypothetical protein (Fig. 2a). Based on these searches, we assessed the expression of each gene with RT-qPCR with positive (250 nM sCSP) and negative (sterile water) controls in a *∆comC* background, incapable of activating *sigX* without sCSP. Upon addition of sCSP, gene expression for most of the highlighted candidates was upregulated (Fig. 2b). Of the genes with unclear involvement in natural transformation, *SCSC_RS09450* (referred to as hyp. prot in the figure) was highly upregulated, whereas both *pheT* and *ciaH* showed lower levels of upregulation. Slight upregulation was also observed in four binding sites located within gene boundaries, namely SCSC_RS05465 (1.30±1.65), SCSC_RS05825 (2.63±1.96), SCSC_RS05935 (2.41±1.96) and SCSC_RS06910 (2.59±2.61).

We utilized these refined search patterns in FIMO to expand the search to 33 genomes of *S. constellatus* [33,35], and identified these genes to be mostly conserved similarly to the binding sites (Fig. 2c). In terms of operon organization, *S. constellatus* appears to share the wider organization of *S. pneumoniae*. Comparing the *S. pneumoniae* D39 genome with *S. constellatus* CCUG 24889, we found the gene *spx* following *cinA* and *recA* in *S. constellatus* (Fig. 2d), whereas it is located elsewhere in *S. pneumoniae*. The gene *spx* is a transcriptional regulator likely involved in stress responses [41]. Downstream of *dprA* in *S. constellatus* one can also identify a second gene, *topA*, which seemingly presents no homologous candidate in *S. pneumoniae*. Similarly, a cinbox was identified upstream a unique hypothetical protein, with no homologous candidate in *S. pneumoniae*, followed by another gene *ccmD* also seemingly not present in *S. pneumoniae*.

### Kinetics of *sigX* expression in *S. constellatus*

**Fig. 3.**
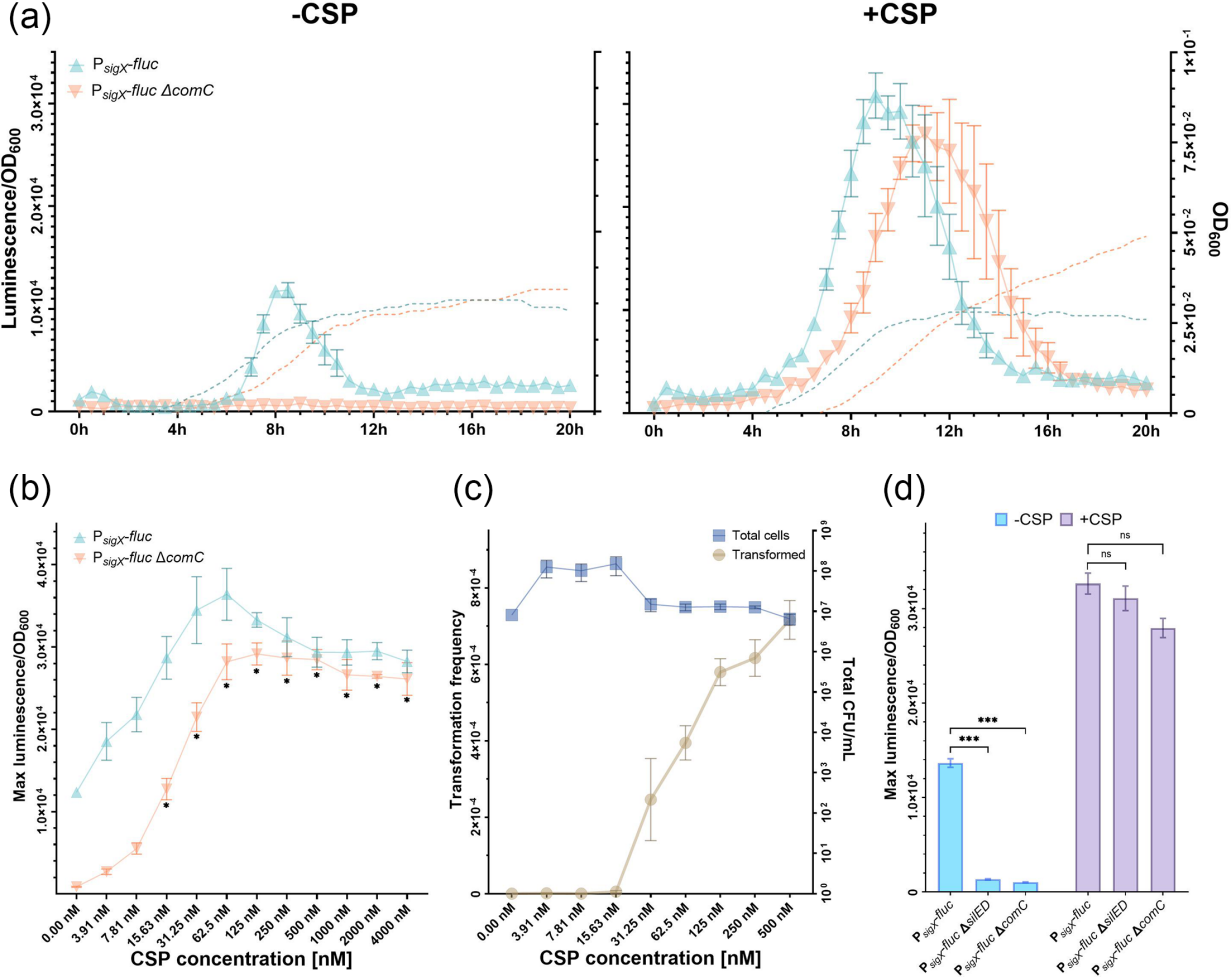
Parameters governing competence and natural transformation. (**a**) Temporal profiles of growth-adjusted luminescence (*sigX* expression) in the P*_sigX_-fluc* and P*_sigX_-fluc* Δ*comC* reporters±CSP. Lines with symbols indicate bioluminescence by the reporter, whereas the dotted lines show the OD_600_ development across time. (**b**) Concentration curve of response to sCSP in the P*_sigX_-fluc* and P*_sigX_-fluc* Δ*comC* reporter. Repeated measures ANOVA and Dunnett’s multiple comparison with the control (0 nM CSP) show a significant (*P<0.05) change in *sigX* promoter activity after addition of 15.6 nM sCSP or more in the P*_sigX_-fluc* Δ*comC* reporter. (**c**) Dose-response of CSP concentration on maximum *sigX* expression and transformation frequency using amplicon DNA as donor. The transformation frequency was calculated by the ratio of CFU counting on selective agar versus the CFU count on non-selective TSB agar. (**d**) Impact of *silED* and *comC* deletions on endogenous and CSP-induced *sigX* expression. Repeated measures ANOVA comparing the *sigX* response in the three mutants, P*_sigX_-fluc*, P*_sigX_-fluc* Δ*silED* and P*_sigX_-fluc* Δ*comC*, with or without sCSP (ns: not statistically significant, ***P<0.001). All data represent mean±sem of three independent biological experiments.

While natural transformation has been traditionally studied in fast-growing streptococci such as *S. pneumoniae* and *S. mutans*, *S. constellatus* exhibits growth dynamics characteristic of the broader anginosus group with a long lag phase, followed by a slower doubling time [42]. This is observed in the laboratory in parallel to clinical conditions, where anginosus species are often observed to develop abscesses and chronic infections [43]. These characteristics require adaptation for the optimization of the existing protocols for competence and transformation studies. As a measurement of competence activity and with the goal of assessing the kinetics under different conditions, we constructed a luciferase reporter system in *S. constellatus* type strain as described previously by Junges *et al*. [24]. For the initial transformation assay of *S. constellatus* type strain CCUG 24889, we adapted from two protocols reported by Bauer *et al*. [17] and Salvadori *et al*. [26]. Briefly, the type strain was grown in TSB medium to OD_600_≈0.04, before 250 nM sCSP and 100 ng amplicon DNA were added in 200 µl aliquots. A number of colonies were obtained after 48 h incubation in anaerobic conditions and a few putative mutants exhibiting the resistant phenotype were genotyped using colony PCR and functionally tested by assessment of luminescence emission (Fig. 3a). We could observe a strong luminescence response in P*_sigX_-fluc* mutant in the presence of sCSP, but also without added peptide, potentially due to endogenous production of CSP. To confirm, a *comC* knockout mutant of the reporter was constructed. This Δ*comC* reporter did not show any activation compared to the background without addition of sCSP (Fig. 3a), indicating that the reporter constructed in the type strain allowed for assessment of both endogenous production and response to exogenous stimuli.

We further sought to reveal the response of *sigX* to different concentrations of sCSP (Fig. 3b) and observed a statistically significant increase in *sigX* expression around 15 nM in the Δ*comC* reporter. While not statistically significant, an induced response was observed as low as 4 nM. The expression plateau was reached with 60 nM sCSP with no additional response on *sigX* expression observed for higher concentrations. Transformation frequency data indicated that a higher concentration of sCSP in the range between 125 and 500 nM yields higher transformation frequencies (Fig. 3c). Such discrepancy may be due to factors involving late recombination gene activation or post-translational events. Finally, as we have identified in the first section of the results and as it has also been reported by others, only one peptide cleavage/export ABC transporter system is present in species of the anginosus group [20,37]. This system is comparatively more similar to BlpAB/SilED than to ComAB (Table S5). To understand if SilED is responsible for exporting CSP in this species and if there are any other redundant transporters, we constructed a *silED* knockout in the *S. constellatus* CCUG 24889 P*_sigX_-fluc* strain. After knocking out *silED*, the endogenous expression of *sigX* was reduced to the same level of the Δ*comC* reporter (Fig. 3d), indicating that SilED is responsible for exporting CSP with no other redundant exporters. In summary, we were able to assess both endogenous and exogenous CSP activity in *S. constellatus*. We further show that concentrations as little as 4 nM already activate the system in this species, and the minimum concentration required for maximum reporter activity is 60 nM. In addition, data indicate that SilED is responsible for exporting CSP in *S. constellatus*.

### Optimizing conditions for competence and transformation

To gain insights into *S. constellatus* transformation and to optimize genetic editing protocols, we aimed to identify conditions that can affect competence development in this species. Optimal conditions for competence development can vary; however, growth medium and cell density have been previously reported as important components that can impact subsequent transformation frequencies significantly [26,44]. As such, assessment of *sigX* activity was performed using the reporter mutant grown in different media – from defined medium C+Y_YB_ and CDM, to complex medium THY, BHI and TSB. The addition of sCSP did not impact reporter activity in C+Y_YB_, CDM or THY, but a difference was observed in TSB and BHI (Fig. S1A). Transformation was tested in all the media listed and recovery of transformants was successful when utilizing TSB, in the conditions tested. It is possible that protocols for each medium would need to be optimized to obtain transformants; nevertheless, based on this finding and on the previous positive experimental outcomes for construction of reporters, we proceeded to use TSB as the method of choice given that it allowed transformation while also yielding a clear distinction in terms of *sigX* activation in the reporter with and without sCSP.

An important parameter in the optimization process was the initial dilution of the preculture. This factor is critical given that streptococci enter competence at a defined cell density [45]. To determine the optimal dilution, precultures frozen at early log phase were diluted 10-, 100- or 1,000-fold in TSB, with or without sCSP, and *sigX* expression was subsequently measured. Significant differences in reporter activity response were found depending on which dilution factor was chosen (Fig. S1B). The endogenous response was seemingly stronger in cultures prepared with lower dilution factors, whereas the response difference obtained from the addition of sCSP is more evident in higher dilutions. Based on these results and previous reports, a dilution factor of 1 : 100 was chosen to further assess the system [44].

**Fig. 4.**
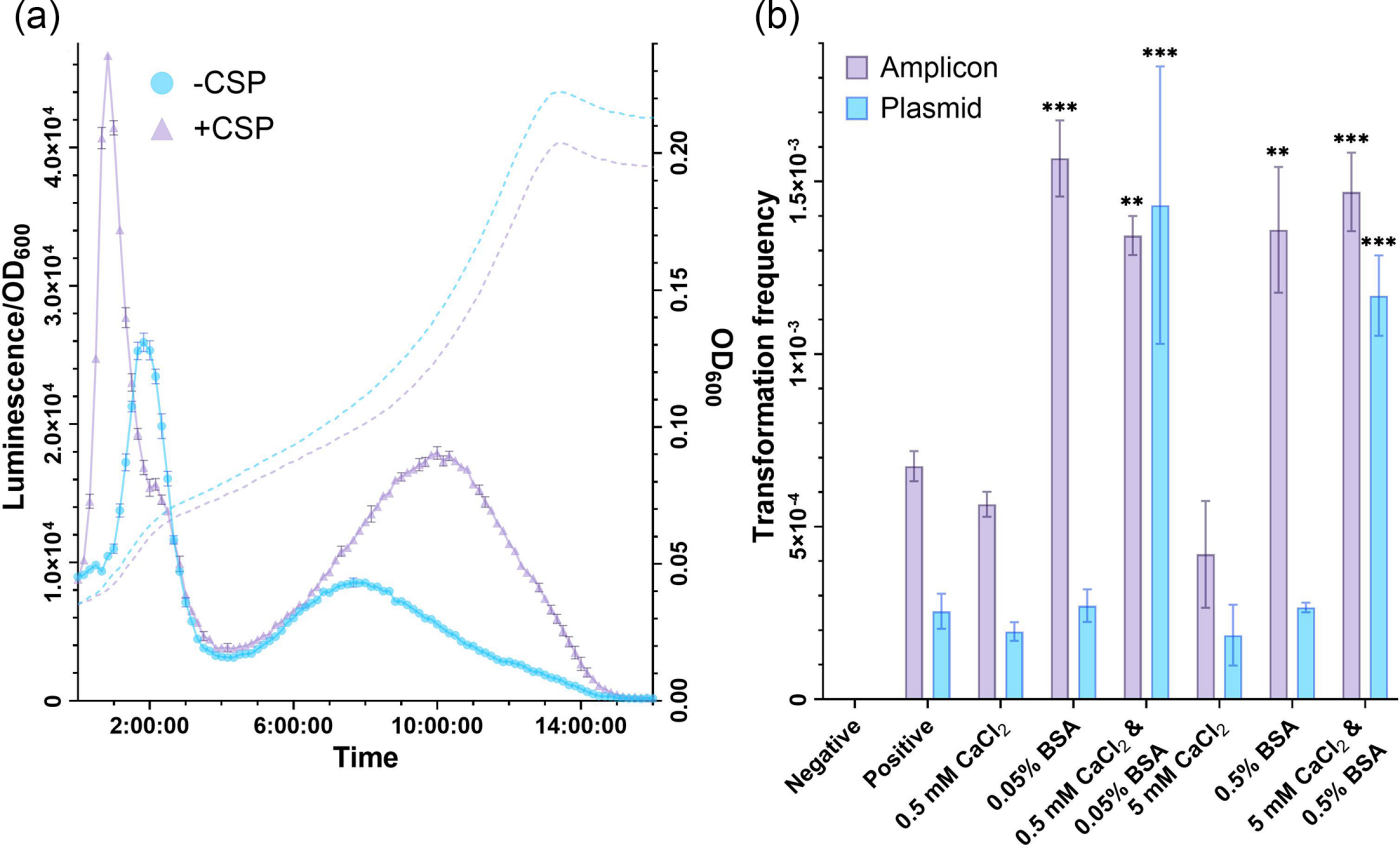
Optimization of the *S. constellatus* transformation protocol. (**a**) Impact of pre-growth inoculum density OD600 ≈ 0.04 on the timing of *sigX* expression. Full lines with symbols indicate bioluminescence over time by the reporter, whereas the dotted lines show the OD_600_ development. (**b**) Effect of BSA and CaCl_2_ on transformation frequencies for amplicon and plasmid DNA. Repeated measures ANOVA with Dunnett’s multiple comparisons against the sCSP-treated (positive) group (**P<0.01, ***P<0.001). All data represent mean±sem of three independent biological experiments.

Maximal *sigX* expression occurred between 6 and 10 h after treatment with sCSP immediately after dilution. Based on our previous experience with transformation of *S. pneumoniae* [24,46], *S. mitis* [44] and *S. mutans* [24,47], we instead allowed the 1 : 100 diluted culture to grow to OD_600_≈0.04 prior to addition of sCSP and DNA, as this approach has previously resulted in significantly improved transformation frequencies. This growth stage typically takes 2–4 h; however, once sCSP is added to a growing culture, peak *sigX* expression is reached within 2 h (Fig. 4a). To further develop the protocol, we asked whether the addition of calcium chloride (CaCl_2_) and BSA, seen to improve transformation likely by assisting in DNA protection/stabilization and DNA-cell association, would affect transformation in *S. constellatus* [24,48, 49]. We compared transformation frequencies in the presence of these compounds utilizing both amplicon and plasmid as donor DNAs. For amplicon DNA, transformation frequencies increased ~2.5-fold when adding 0.05% BSA (Fig. 4b) compared with the positive control of 250 nM CSP treatment alone, but no discernible difference in combination with 0.5 mM CaCl_2_ was observed. For transformation with plasmid DNA, addition of a mix of 0.05% BSA and 0.5 mM CaCl_2_ increased the transformation frequency ~2-fold compared with the positive control, while any component on its own did not significantly change transformation yields. No significant increase in transformation frequency was observed when increasing concentrations to 5 mM CaCl_2_ or 0.5% BSA. As such, the optimized conditions for transformation were found to be with addition of 0.05% BSA and 0.5 mM CaCl_2_ when transforming with plasmid DNA and 0.05% BSA when utilizing amplicon DNA. In conclusion, we identified conditions in the protocol to transform *S. constellatus* that increase transformation frequencies significantly. Further, we have successfully constructed mutants in all anginosus species utilizing this protocol (data not shown). Of note, while the length of the homogenous flanking loci can affect the transformation frequency [47], we did not observe any difference in transformation yields between a 3.5 kbp and a 7 kbp amplicon from the same genetic background. As such, most of the results presented are results of transformation with the shorter amplicon.

### The effect of environmental stressors on natural transformation

**Fig. 5.**
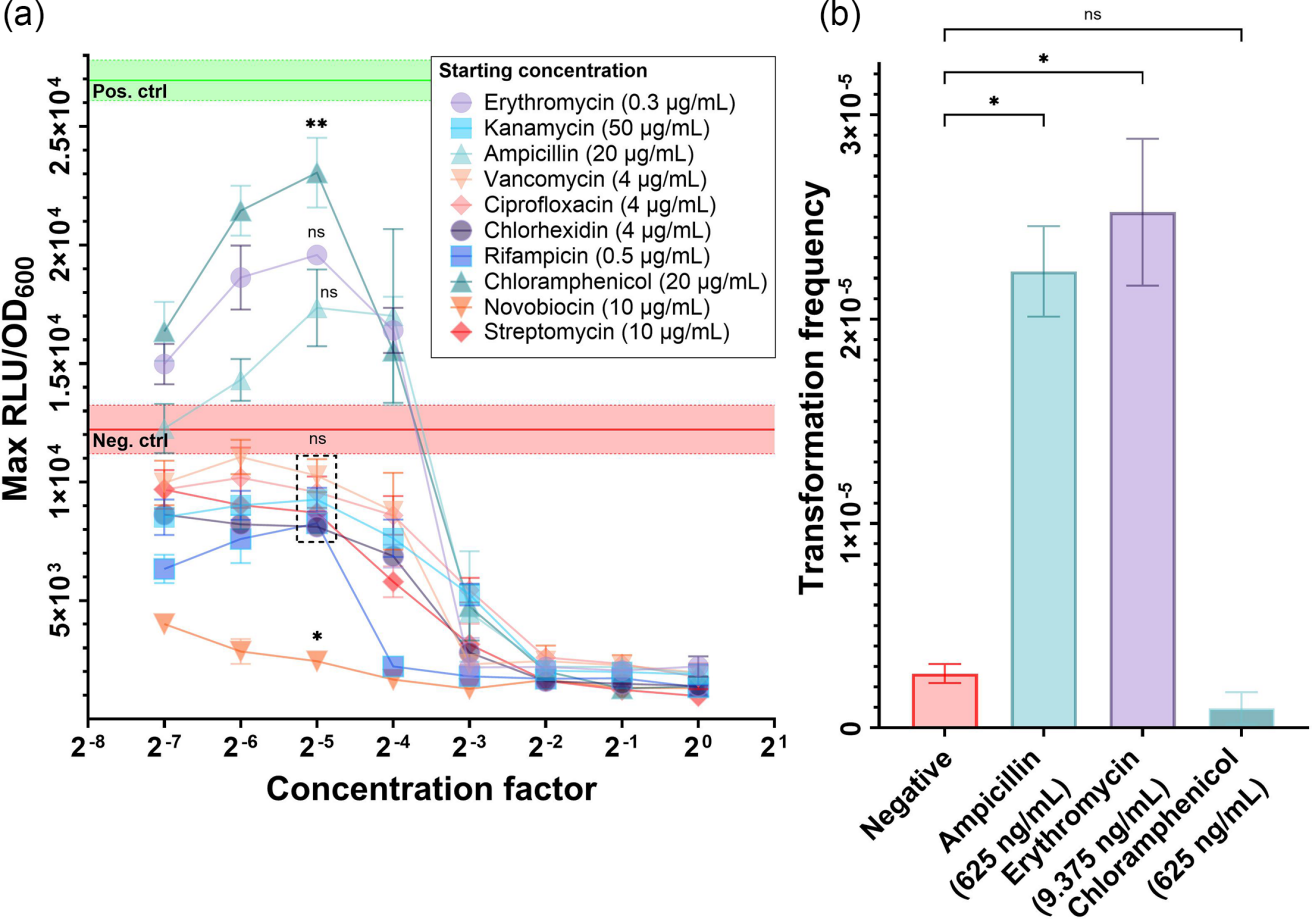
Response of *sigX* expression to antibiotic and antiseptic compounds. (**a**) Maximum growth-adjusted luminescence of the *sigX* reporter strain (SC003) exposed to various antibiotics and antiseptics at inhibitory and subinhibitory concentrations. Green and red horizontal lines indicate negative (water) and positive (250 nM CSP) controls. ANOVA followed by Dunnett’s multiple comparisons test of the different antibiotics at 2^−5^ concentration factor against the negative control (no treatment) indicate a significant (ns: not statistically significant, *P<0.05, **P<0.01) difference for novobiocin (312.5 ng ml^−1^) and chloramphenicol (625 ng ml^−1^). Erythromycin (9.375 ng ml^−1^) was borderline with an adjusted *P*-value of 0.08. (**b**) Following reporter assay analysis, all antibiotics which increased the activation of *sigX* were tested using transformation assays. ANOVA analysis and multiple comparisons Dunnett’s test show a significant (*P<0.05) increase in transformation frequency in the presence of ampicillin and erythromycin, when compared with the negative control. All data represent mean±sem of three independent biological experiments.

**Fig. 6.**
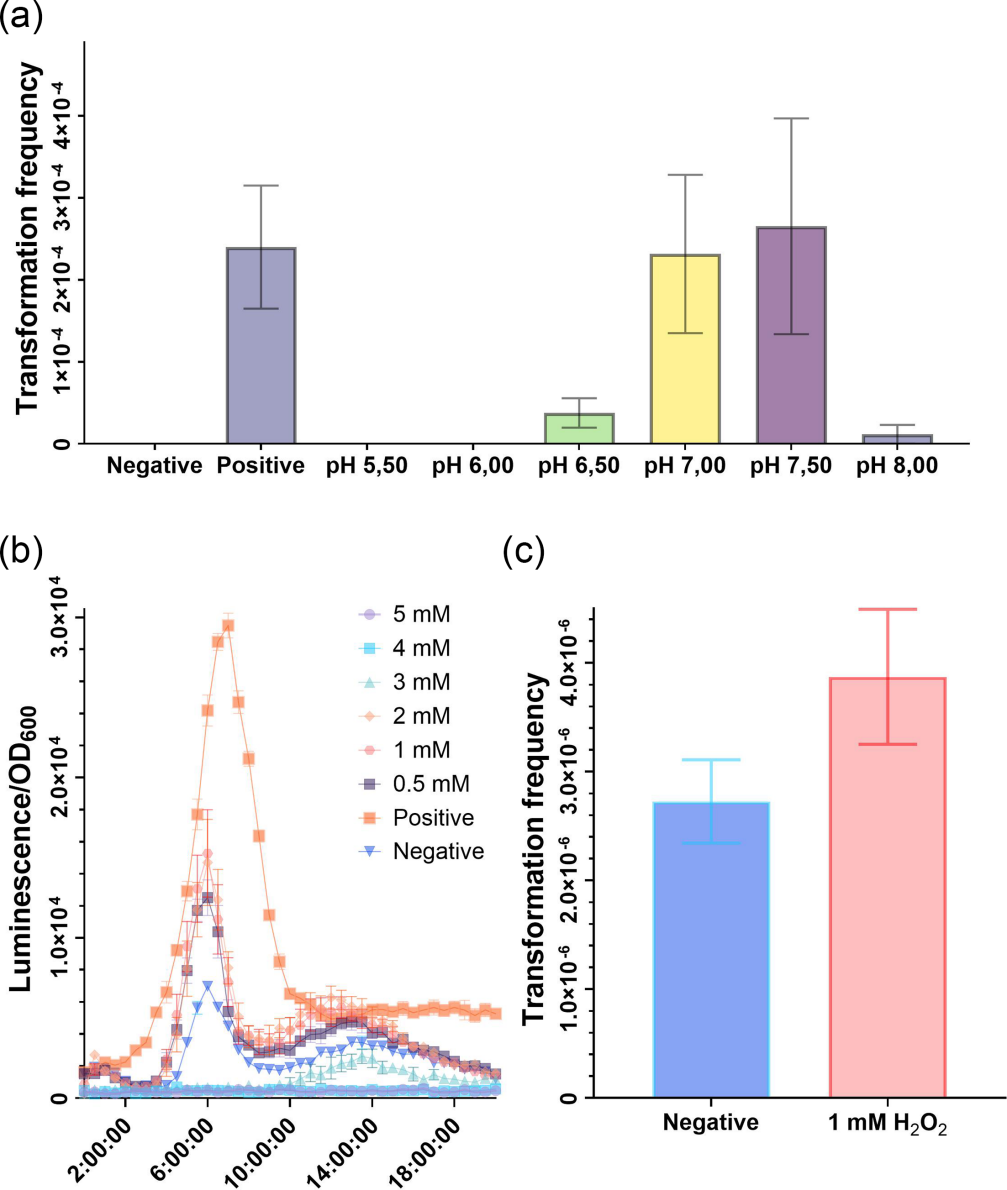
Environmental stressors effect on transformation and competence. (**a**) Effect of pH on transformation frequency in the type strain CCUG 24889. (**b**) Dose-dependent effect of growth-adjusted luminescence by hydrogen peroxide. Negative (water) and positive (250 nM CSP) controls are represented. Using repeated measures ANOVA and Dunnett’s test, no significant increase in *sigX* promoter activity was identified when compared with the negative control at the maximum activity 6 h after experiment initiation. (**c**) Transformation frequency incubating with 1 mM hydrogen peroxide. Despite an increase, no statistical significance was observed in transformation frequencies between the negative control and treatment with 1 mM H_2_O_2_, with a paired t-test. All data represent mean±sem of three independent biological experiments.

Environmental stressors such as antibiotics have been shown to influence HGT and affect bacterial ability to enter the state of competence [50,52]. As the anginosus group of streptococci thrives at a variety of body habitats and challenging growth conditions such as tumor microenvironments and abscesses [53,55], we hypothesized that *S. constellatus* could present a distinct ability to respond to environmental stressors by activating the natural competence system to gain a survival advantage in such conditions. We tested a range of antibiotics at different concentrations and identified that ampicillin, chloramphenicol and erythromycin generated an increased *sigX*-expression consistently at multiple subinhibitory concentrations (Fig. 5a). The strongest effect on *sigX* expression was observed at 625 ng ml^−1^ for chloramphenicol and ampicillin, while erythromycin had its strongest response at 9.4 ng ml^−1^. Most of the other antibiotics tested did not seemingly induce *sigX* expression, and in some cases, e.g. novobiocin, seemed to show a repressive effect. For ampicillin and erythromycin that upregulated the system, we also observed significantly increased transformation frequencies relative to the negative control, whereas transformation frequency did not appear to change with exposure to chloramphenicol (Fig. 5b). In conclusion, subinhibitory concentrations of ampicillin and erythromycin induced competence expression in *S. constellatus* and led to increased transformation frequency. Further, as *S. constellatus* is often isolated from acidic and inflammatory environments, we tested transformation in a wide range of pH between 5.5 and 8.0 and were able to recover transformants between 6.5 and 8.0, with the highest frequencies between 7.0 and 7.5, as expected (Fig. 6a). H_2_O_2_ is a reactive oxygen species and an important component in species–species competition of streptococci, as well as a potent antimicrobial component of the immune response. After exposure to subinhibitory concentrations of H_2_O_2_, we observed tolerance up to 3 mM H_2_O_2_, and an elevation of *sigX* expression at 0.5, 1 and 2 mM (Fig. 6b). Additionally, higher transformation frequencies were also obtained in the presence of 1 mM H_2_O_2_ compared to the negative control (Fig. 6c), although this difference was not statistically significant. Altogether, these data indicate *S. constellatus* can adapt to diverse environmental conditions while maintaining activation of its competence system, which may provide competitive advantage under high-stress environments.

## Discussion

HGT plays a central role in streptococcal adaptation, yet information on the mechanistic basis of natural transformation within the anginosus group remains limited. Although *S. constellatus* has previously been reported to undergo natural transformation, its regulatory circuit and genetic competence determinants have not been systematically characterized. In this study, we demonstrated that most *S. constellatus* strains harbor a complete competence regulon, including the core genes required for DNA uptake (*comFA/C, comEA/C*, *comG, endA*) and homologous recombination (*cinA*, *cclA, recA, coiA*, *radA*, *dprA*, *ssbB*). We have also shown that *S. constellatus* relies on the bacteriocin exporter SilED for export of CSP. As adaptation to environmental challenges is clinically relevant for SAG, upregulation of the system and consequently an enhanced capability to transform was observed for *S. constellatus* in a variety of stress scenarios. Notably, subinhibitory antibiotic concentrations of erythromycin, ampicillin and chloramphenicol consistently activated *sigX* expression. Finally, with an optimized protocol, we increased transformation frequencies significantly, which further facilitates the construction of mutant strains and deepens our understanding of HGT in this important opportunistic pathogen.

While the interplay between competence and bacteriocin production is common among streptococci, the complete absence of ComAB and consequently reliance on SilED to activate competence seems to be a unique feature of the anginosus group. While ComAB is generally conserved across all pneumococcal strains and important for competence regulation, about a quarter of *S. pneumoniae* strains also encode an intact BlpAB, analogous to SilED [37]. Interestingly, it has been shown that in BlpAB-positive strains of pneumococcus, the BlpAB transporter can mediate the secretion of both bacteriocins and CSP, suggesting that these strains presenting BlpAB could activate competence independently of ComAB [37]. In addition, Kjos *et al*. have demonstrated that when BlpAB is disrupted, ComAB can compensate by exporting BlpC [56]. These findings support the notion of redundant function between the transporters ComAB and BlpAB, as observed in our results indicating that SilED is essential for exporting CSP in *S. constellatus*. In the pneumococcus, it was further observed that strains containing both active transporters seem to have a competitive advantage, likely acting as aggressors to outcompete other strains and species [37]. Other streptococcal species such as *Streptococcus thermophilus* and *Streptococcus gallolyticus* have been shown to contain functional BlpRH systems that regulate bacteriocin production through quorum sensing [32,57]. The presence of a single transporter in SAG can also have implications for its survival against other species in human microbiomes, and further research into competition assays in clinically relevant scenarios is warranted.

No ComW orthologs were identified in *S. constellatus* in this study. In *S. pneumoniae*, ComW seemingly presents two main functions: stabilizing SigX and enhancing its ability to form an active RNA polymerase holoenzyme complex, possibly by assisting SigX to successfully latch onto RNA polymerase, which is necessary for late gene activation [10]. It has also been shown that ComW binds to DNA, although not in a sequence-specific way, and this DNA-binding activity appears essential for its function in transformation [58]. While *comW* seems to be conserved across species of the mitis group, including *S. pneumoniae*, *S. mitis* and *S. oralis*, previous reports have not identified orthologs in *S. anginosus* [17]. This goes in line with the findings we presented here regarding *S. constellatus* genomes. We did, however, notice one study reporting a possible *comW* in *S. anginosus* SK1138 [58]. We expanded the search to other anginosus species, namely *S. intermedius* and *S. anginosus*, utilizing the SK1138 gene and identified a total of five strains of *S. anginosus* with an ortholog *comW*. No matches had been obtained with the *comW* gene from *S. pneumoniae* D39. Pairwise comparison of the ComW proteins in *S. pneumoniae* D39 and *S. anginosus* SK1138 showed 40% identity and 57.5% similarity. As a follow-up, homology searches across the domain of bacteria, excluding SAG species, showed a close similarity between the *comW* variants found in the SAG species and the variants found in four different strains of *Streptococcus cristatus*. The genomic locus of *comW* in *S. pneumoniae* is in the neighboring regions of a cluster of rRNA close to the chromosomal origin of replication. In contrast, the *comW* identified in *S. anginosus* is located more distantly from the origin of replication, and not near any rRNAs. Importantly, this organization resembles the genomic context of *comW* found in *S. cristatus* where it is located close to a cluster of glycosyltransferases. While the investigation into the mechanistics of *comW* in this strain was outside the scope of this study, we were able to recover transformants in one of the *comW*(+) strains with similar yields compared to *comW*(-) strains (data not shown). Further, it would be important to assess whether *comW* is indeed functional in these strains, or whether it is a byproduct of previous HGT events. Finally, another study in *S. pneumoniae* identified mutations in the primary sigma factor, encoded by *rpoD*, that would bypass the need for *comW* [59]. However, such mutations were not present in the *S. constellatus* genomes assessed in this study. Utilizing our homology approach, we were not able to identify any other potential candidate that could functionally replace *comW* in *S. constellatus*. It would be plausible to hypothesize that there are yet unidentified patterns of mutations in the anginosus group that would bypass the need for ComW, as observed for a subset of pneumococcal strains.

Higher transformation frequencies for amplicon DNA as opposed to plasmids were observed in our study, which goes in line with previous reports assessing natural transformation efficiency in streptococci [44,47]. During transformation, EndA, an endonuclease localized in the membrane [60], degrades one strand for the uptake of a ssDNA molecule. RecA and other single-stranded proteins then facilitate recombination into the chromosome based on sequence homology [54]. For plasmids, such process then requires the entry of multiple single-stranded plasmid DNA fragments, which will then require reassembly [61]. Although size and divergencies in specific plasmid and amplicon sequences can play a role, generally, linearized DNA transformation is considered as a more direct process for uptake and recombination, showing thus the higher frequency rates of transformation observed. For the SigX-regulon, based on the identification of binding sites, while the organization in *S. constellatus* is generally similar to *S. pneumoniae*, there were a variety of genes or ORFs with no orthologs identified and with unknown functions in the competence development process. These could be targets for further investigation and the strategy we employed for the motif-pattern search may be applicable in other bacteria, as most of the targets identified showed upregulation in the system upon sCSP treatment. Finally, the coupling with transcriptome data may be a useful resource in such cases as well as the assessment of direct SigX binding to the hypothesized promoter sites. Whole sequencing of transcripts or chromatin immunoprecipitation sequencing would also allow for detection of any atypical binding sites missed by our motif-based approach.

A recent study confirmed the sequence of the *S. constellatus* CSP as a 16-amino acid peptide (DSRIRMGFDFSKLFGK) and explored the residue significance via the construction of peptide analogues [18]. With structure-activity assays using alanine and d-amino acid scans, the authors revealed that the N-terminal region is vital for activation of the system and, notably, the C-terminal region is also crucial for *S. constellatus*. As such, they were able to identify the analogue CSP I4A to be a more potent activator of the competence system. Potency was measured by the proportional bioluminescence estimated by the EC50 of a *S. constellatus* P*_sigX_::fluc ΔcomC* reporter strain to different concentrations of the CSP isoforms. However, this increased response and potency was not translated into improved transformation frequencies. Further, the identification of CSP in supernatants indicates that the endogenous activation of *comC* coupled with the following cleavage/export of the mature peptide is functional in this species. Also similar to our findings was their discovery that low concentrations of CSP were observed to upregulate the system in their reporter system.

A further key finding by Mehrani *et al*. was that the competence regulon had no role in biofilm formation, in contrast to previous observations in other streptococci [18,62, 63]. Increased biofilm formation is likely due to cell lysis and release of eDNA. On this note, we identified that the murein hydrolase LytF was present in all genomes surveyed of *S. constellatus* as part of the SigX regulon. Despite copies of LytF being identified in a variety of streptococcal species, e.g. *S. gordonii*, *Streptococcus sanguinis*, *S. infantarius* and *S. cristatus*, their structure and loci can diverge across species [13]. While *S. gordonii* DL1 has been shown to possess a LytF with three BSP domains followed by a CHAP domain, *S. sanguinis* SK36 presents a larger gene with five BSP domains [64]. The structure and loci of the LytF in *S. constellatus* is similar to *S. sanguinis* SK36. Interestingly, a recent study in SK36 reported findings supporting the role of LytF in extrusion of the transformation pilus across the cell wall, and not directly on fratricide [65]. The authors also investigated the role of a CAAX protease as a potential LytF-immunity gene but found no evidence of increased lysis in knockout mutants. In *S. constellatus*, this protease is located in the Sil locus and named *silX*. Its function has been suggested to involve the export and processing of the peptide SilCR, while no immunity has been detected towards angicin [66], a bacteriocin identified in *S. anginosus* BSU 1211. In *S. mutans*, it has been recently reported that LytF plays a role in the release of cytoplasmic membrane vesicles via cell wall degradation [65], also resulting in lysis of a subpopulation of cells [67]. One interesting observation in our study was the absence of the typical growth arrest following sCSP addition, which is generally attributed to lysis of a subpopulation of cells during competence [12], and under the conditions tested, this was not observed in *S. constellatus*. This lack of detectable lytic activity may support the previous findings in *S. sanguinis*, suggesting that the lytic potential of *lytF* could be condition dependent.

Environmental stressors have been shown to influence competence in a variety of bacterial species including *S. pneumoniae* and *Legionella pneumophila* [52,68]. In *S. constellatus*, we identified that subinhibitory concentrations of erythromycin, chloramphenicol and ampicillin induced *sigX* expression, with increased transformation rates observed with erythromycin and ampicillin (Fig. 5b). Antibiotics that target protein synthesis, such as erythromycin and chloramphenicol, can lead to a higher number of misfolded proteins, which in turn can reduce CSP degradation extracellularly given the HtrA protease may be saturated processing these misfolded proteins [23]. As such, the stronger activation of *sigX* in these two cases may be due to a higher concentration of CSP extracellularly; however, it is interesting that higher transformation rates were observed for erythromycin but not chloramphenicol. The lack of effect on transformation rates despite increased *sigX* expression has been observed previously in the case of erythromycin and tetracycline [69]. The discrepancy between gene expression and phenotype observed by us and others could be attributed to different hypotheses, such as an uncoordinated general stress response after exposure to antibiotics [70,71], or aberrant translation patterns for bacteria, particularly in the presence of drugs that can affect protein synthesis [72,73]. While ampicillin was not seen to stimulate competence previously in *S. pneumoniae* [52,69], we observed an inducing effect in *S. constellatus*. As β-lactams interfere with the cell wall, it is a possibility that the presence of ampicillin at subinhibitory concentrations can lead to pore formation facilitating the translocation of molecules between intra and extracellular environments. As antibiotics that affect DNA replication elongation, e.g. novobiocin and ciprofloxacin, have been shown to induce competence via an increase of the *comCDE* gene dosage that are located close to the replication origin in *S. pneumoniae*, we also expected to see induction of *sigX* by these compounds [74]. However, no stimulation was observed with a seemingly repression of *sigX* when cells were exposed to novobiocin. In addition, while kanamycin has been shown to be a potent inducer of competence in *S. pneumoniae*, no *sigX* upregulation was observed in our study [74]. As antibiotics acting through similar pathways can affect streptococci divergently, this supports the notion that their effect may be potentially species- and condition-dependent. Finally, it is important to consider that the induction of the competence regulon may provide benefits that improve bacterial survivability and are independent of phenotypical transformation [75,76], thus providing a competitive advantage to competent subpopulations regardless of DNA uptake.

Further research is warranted to better unravel the dynamics of HGT at a community level under stress. Important variables to consider are available extracellular DNA, CSP gradients and patterns of competence activation in biofilms. Previous studies assessing transformation efficiency at various DNA concentrations have shown nearly linear increases in efficiency corresponding with increases in donor DNA at lower concentrations until a plateau is reached [24,38, 44, 47]. For the assays presented here, we have assessed transformation in planktonic cultures utilizing saturating concentrations of DNA for both amplicon and plasmid transformations. As it has been shown that close cell-to-cell contact in biofilms and in filter mating leads to a higher frequency and size of recombination in *S. pneumoniae* [77], it would be plausible to consider that rates of competence activation and natural transformation would be even higher for *S. constellatus* when grown in biofilms. In this context, the high responsiveness of this species to low doses of CSP might be a good indicator of *in vivo* functional activity. Still, sequence homology, DNA complexity, and the presence of nucleases in polymicrobial biofilms could potentially affect DNA availability and transformation rates, and, thus, should be examined further.

Altogether, the findings of this study expand our knowledge on natural transformation in streptococci and further facilitate the study of molecular mechanisms of pathogenicity in the anginosus group through the development of a genome editing protocol with higher transformation frequencies. In addition, data on environmental stressors influencing the rate of HGT is particularly relevant for the study of adaptability and survivability in this group of species. These findings may also carry implications clinically as (i) these species can be exposed to a variety of antibiotic formulations and concentrations as part of the commensal microbiota and (ii) some of these antibiotics can be recommended for the treatment of streptococcal infections. For instance, β-lactams and ampicillin are part of the line of treatment against infective endocarditis caused by viridans group and other types of streptococcal infections [78,81]. Thus, understanding the mechanisms through which different antimicrobial drugs and formulations within the same class and mode of action divergently affect bacterial physiology and resistance rates is a priority.

## Supplementary material

10.1099/mic.0.001726 Supplementary Material 1.

## Abbreviations

BHI: Brain-Heart Infusion
CDM: chemically defined medium
CFU: Colony-Forming Units
Cq: quantification cycle
CSP: competence-stimulating peptide
FIMO: Find Individual Motif Occurrences
HGT: horizontal gene transfer
MEME: Multiple Em for Motif Elicitation
NCBI: National Center for Biotechnology Information
RLU: Relative Light Units
RT-qPCR: Reverse Transcript quantitative Polymerase Chain Reaction
SAG: *Streptococcus anginosus* group
sCSP: synthetic competence-stimulating peptide
Sil: Streptococcus invasion locus
THY: Todd-Hewitt Broth with Yeast Extract
TSB: Tryptic Soy Broth
