# Supplementary material for: Genetic and functional characterization of the natural transformation system in Streptococcus constellatus
Source: Microbiology (Reading). 2026 Jun 18;172(6):001726. doi: 10.1099/mic.0.001726 (PMC13278378; doi:10.1099/mic.0.001726)
Supplement: Supplementary Material 1. [file mic-172-01726-s001.pdf]

Table S1. Bacterial strains, peptides, and antibiotics used in the study.

Strain	Description	Source
CCUG 24889^T	*Streptococcus constellatus* subsp. *constellatus* CCUG 24889	Culture Collection University of Gothenburg
SC002	CCUG 24889^T, but pRJ11	This study
SC003	CCUG 24889^T, but P_{sigX} -*fluc-aad9* (spc^R)	This study
SC005	SC003, but Δ*comC* ::*ermB* (erm^R)	This study
SC011	CCUG 24889^T, but Δ(SCSC_RS06260 – SCSC_RS06255)::*aphA-3* (kan^R)	This study
SC013	CCUG 24889^T, but Δ*comC::ermB* (erm^R)	This study
SC015	CCUG 24889^T, but Δ*silED* ::*ermB* (erm^R)	This study
SC016	SC003, but Δ*silED* ::*ermB* (erm^R)	This study
SC017	SC003, but Δ(SCSC_RS06260 – SCSC_RS06255)::*aphA-3* (kan^R)	This study
**Peptide**	**Sequence**	**Source**
CSP	DSRIRMGFDFSKLFGK	GenScript, Piscataway, USA
**Antibiotics**	**Standard concentration**	**Manufacturer**
Spectinomycin	500 µg/mL (selective plates)	Sigma-Aldrich
Kanamycin	500 µg/mL (selective plates) / 50 µg/mL (reporter assay)	Sigma-Aldrich
Erythromycin	10 µg/mL (selective plates) / 0.3 µg/mL (reporter assay)	Sigma-Aldrich
Ampicillin	20 µg/mL (reporter assay)	Sigma-Aldrich
Tetracycline	5 µg/mL (selective plates)	Sigma-Aldrich
Vancomycin	4 µg/mL (reporter assay)	Sigma-Aldrich
Ciprofloxacin	4 µg/mL (reporter assay)	Sigma-Aldrich
Chlorhexidin	4 µg/mL (reporter assay)	Sigma-Aldrich
Rifampicin	0.5 µg/mL (reporter assay)	Sigma-Aldrich
Chloramphenicol	20 µg/mL (reporter assay)	Sigma-Aldrich
Novobiocin	10 µg/mL (reporter assay)	Sigma-Aldrich
Streptomycin	10 µg/mL (reporter assay)	Sigma-Aldrich

Table S2. Donor DNA for transformation assays and oligonucleotides used in the study.

Donor DNA	Description	Size	Primers
aSC011.3	CCUG 24889^T Δ(SCSC_RS06260 – SCSC_RS06255)::*aphA-3* (kan^R)	3409 bp	RJ76-77
aSC011.7	CCUG 24889^T Δ(SCSC_RS06260 – SCSC_RS06255)::*aphA-3* (kan^R)	7097 bp	RJ108-RJ109
pRJ11	pDL278, but *aad9* (spc^R)::*aphA-3* (kan^R)	7646 bp	

Primers	Description		5'-oligonucleotide-3'
RJ23	SC003	F1 of SC003	AGGAAATGATGAGCGCGAAC
RJ24		F1 of SC003	atttacctcctcgaggatccCTTGAAGTCCATTTCATAACCC
RJ25		F2 of SC003	gttatgaaatggacttcaagGGATCCTCGAGGAGGTAAATG
RJ26		F2 of SC003	tactagtacagctagtggttCGCGCTTACCAATTAGAATG
RJ27		F3 of SC003	cattctaattggtaagcgcgAACCACTAGCTGTACTAGTAG
RJ28		F3 of SC003	TTTACGATCCGAAAACCTTC
RJ29		Nested of SC003	GATGTGAAGGGTCGTGAAGC
RJ30		Nested of SC003	AGATTCCCTACTGCTGCCTC
RJ60	SC005	F1 of SC005	CGTACAATGATGGGCTGAATAATC
RJ61		F1 of SC005	tttgggcccgGCTAAAAATTTTTTTCATAAATTCTCCTTAATG
RJ62		F2 of SC005	aatttttagcCGGGCCCAAAATTTGTTTGATTTG
RJ63		F2 of SC005	aatcaaatccGCGACTCATAGAATTATTTCCTCC
RJ64		F3 of SC005	tatgagtcgcGGATTTGATTTTTCTAAACTTTTTGG
RJ65		F3 of SC005	CTTTTTGCTCAGTTTGGGTTTTATTATATC
RJ66		Nested of SC005	AGGATTTGTTTGGATGTCATTG
RJ67		Nested of SC005	GCACCCTTTATGAATGCTTAGT
RJ68		Detect comC	GCAAAAAGGAAATAACTAAAGTGGA
RJ69		Detect comC	TCAAATCCCATTCTTATTCGAC
RJ70	SC011	F1 of SC011	TTCAAGTGCGGGGAAATTG
RJ71		F1 of SC011	aaacggcgcgATGATTATCAGCAAGTTTTTCTTG
RJ72		F2 of SC011	tgataatcatCGCGCCGTTTGATTTTTAATG
RJ73		F2 of SC011	ctttttctccCCGGCCATCGATACAAATTC
RJ74		F3 of SC011	cgatggccggGGAGAAAAAGAGGAGCTAAAAATAAC
RJ75		F3 of SC011	AACAGAGCACAAGTTGAAC
RJ76		Nested of SC011.3kb	GAAATTGGGAAGAtAGcATTTGG
RJ77		Nested of SC011.3kb	AATGATAATCGTTTCATTGTCTGC
RJ108	SC011	Nested of SC011.7kb	CCTTTTGTCGGTACATAAGAAGA
RJ109		Nested of SC011.7kb	CTCAGAAATTAGCAACCATTATGC
RJ110	SC016	F1 of SC016	CTGCCAGAGAATAATATGC
RJ111		F1 of SC016	tttgggcccgGCAATCTCTTGCATCTATTTG
RJ112		F2 of SC016	aagagattgcCGGGCCCAAAATTTGTTTG
RJ113		F2 of SC016	tgacaatagtGCGACTCATAGAATTATTTCCTC
RJ114		F3 of SC016	tatgagtcgcACTATTGTCATTGCGAAAAAATC
RJ115		F3 of SC016	GATATCGCAGCTTTTCTTAC
RJ116		F4 of SC016	TTTATCCACCCCAGCGAAC
RJ117		F4 of SC016	AACTTTATAGTAAGGTTGGTATCTGGA
RJ128	*coiA*	F	AAGGAAGAGTCATGCGACCG
RJ129		R	GCCCAAGTAAACAAGGCAGC
RJ130	*ciaH*	F	ACTCTTTGTCAGCTGTTCCACT
RJ131		R	TCAAGTTATGCGCTCAAGCC
RJ136	*cclA*	F	GCAACGCAGCCGATTGATAA
RJ137		R	TGGTCGTTGATCGCTTTCCT
RJ140	*comEA*	F	ATCCAAAAAGCGGGTGGACT
RJ141		R	ATTCCCTTTTTGTGCCGCAG
RJ142	*dprA*	F	TCAATTCCGCGTGCTAAACC
RJ143		R	TAGCCGAGTAGGCAGTCAATC
RJ144	SCSC_RS09450 / hyp. prot.	F	TCTGACACAATCTTCATCTTTTGC
RJ145		R	ACAGAAGTCATCACCGTTTTCG
RJ146	*comGA*	F	GGCGTTCATCTCCAATTCGC
RJ147		R	GGAAGAACGAGCACAGGACA
RJ148	*ssbB*	F	CGGCTAATTTGCCCCAAACA
RJ149		R	GCAACGCTTGCTGTTAATCG
RJ150	*cinA*	F	CCAGTCGACTCGCGAAGAAA
RJ151		R	GTGGCTTGGGTCCAACAGAA
RJ156	*comFA*	F	GCAGTTGCCAAGCGAAAAGA
RJ157		R	TCGCCATGCAGGAGTGAAAT
RJ160	*dut*	F	GGCGCAATAACTGTACGCTC
RJ161		R	TACTGCCAAAGCGTGAGACG
RJ164	*pheT*	F	TAGCATATGAGGTGGCAGCG
RJ165		R	ACGCACTGCATAAAAAGGCG
RJ168	*radC*	F	TTGCCAAGAGAACGTTTGGT
RJ169		R	GCCAGTGCGGATAAGAATGG
RJ190	*gyrA*	F	GCACGAGCTTCTGCCTTTTC
RJ191		R	TCCTAAAATTCTCTCCTTGCGTGA
RJ204	*lytF*	F	GCAGCATAATTGGGTTGAAAGG
RJ205		R	CTGGTCTGAAGTCAACGGGC
RJ206	*comD*	F	TCCCTCGCCCTTTGTTGAAA
RJ207		R	TGCCATAGAAGGAGCAGCAG
RJ208	*sigX*	F	TTGGGACCAAGAAGGGATGC
RJ209		R	GCTTTCTTGTTTGCGCAGGA
RJ210	*murB*	F	CAAAACCGCGAATACCACCG
RJ211		R	GGTGGAGCTGCCGATTATCT
RJ214	*spx*	F	CATTACGAACCGAGCGAGGTA
RJ215		R	TGGGGTTGCAAGTATTGTGTCT
RJ216	SCSC_RS05470 (ORF-3)	F	GTGAAAGAGGCGAGCGTTTG
RJ217		R	AGAGTTAGCGAGGCGCTTTC
RJ218	SCSC_RS05830 (ORF-1)	F	TGAGTTCGCCAATGGAAGTGT
RJ219		R	ATTGGTCGGTGCGTGGTTTA
RJ220	SCSC_RS05940 (ORF-3)	F	ATACCCACATGGCACACCAG
RJ221		R	CCAATGGAGGTATCGGAGGC
RJ222	*murC* (ORF-2)	F	GGAACCCGTCGTAGAACGAA
RJ223		R	ACAAGGAGTTGCGTCGGATT
RJ230	*ciaR*	F	AATAATCGTCGGCTCCCAGC
RJ231		R	TACGAAGCCGAAAGTGGTGT

UPPERCASE: Annealing region, lowercase: overlapping region.

Table S3. Genomes utilized in the study.

Species	Sub-species	Strain	Genome size [bp]	G + C [%]	CDS	rRNA	Pseudogenes	Level	#	N50 (kb)	Coverage	Type assembly coverage	*sigX* count	Accession
S. constellatus	*constellatus*	SK53	1 840 061	38	1 715	38	80	Contig	54	70.2	28.66x	98.53%	0	GCF_000257785.1
S. constellatus	*pharyngis*	C232	1 935 414	38	1 790	74	127	Complete	1	1935.4		97.38%	3	GCF_000463395.1
S. constellatus	*pharyngis*	C1050	1 991 156	38	1 839	74	127	Complete	1	1991.2		96.78%	3	GCF_000463425.1
S. constellatus	*pharyngis*	C818	1 935 662	38	1 787	74	129	Complete	1	1935.7		97.39%	3	GCF_000463445.1
S. constellatus	*pharyngis*	SK1060	1 950 566	38	1 807	53	159	Contig	66	143.3	35x	99.63%	1	GCF_000474135.1
S. constellatus		KCOM 1650	1 965 746	38	1 837	49	80	Contig	5	1200.0	1331x	86.32%	1	GCF_000814045.1
S. constellatus		317_SINT	1 853 071	38	1 712	54	86	Contig	48	85.8	29x	88.25%	3	GCF_001072275.1
S. constellatus		783_SANG	1 913 462	38	1 809	37	85	Scaffold	69	77.4	21x	85.39%	1	GCF_001074375.1
S. constellatus		925_SCON	2 043 273	38	1 965	35	101	Contig	124	46.3	22x	86.39%	1	GCF_001075725.1
S. constellatus		KCOM 1039	1 885 802	38	1 728	71	83	Scaffold	2	1900.0	2258.9x	86.57%	3	GCF_003570855.1
S. constellatus	*constellatus*	ATCC 27823	1 867 902	38	1 764	49	85	Scaffold	42	111.3	7300x	97.98%	3	GCF_008633005.1
S. constellatus	*constellatus*	13-11-14	1 800 973	38	1 698	57	79	Scaffold	16	933.5	3700x	84.89%	3	GCF_008633025.1
S. constellatus	*pharyngis*	15-01-28	1 914 344	38	1 791	62	125	Scaffold	13	275.6	600x	96.75%	3	GCF_008633405.1
S. constellatus		D6t1_180914_C10	1 863 546	38	1 756	43	90	Scaffold	85	39.8	33.32x	84.35%	1	GCF_015555235.1
S. constellatus		1001254J_160919_C10	1 755 446	38	1 627	48	82	Scaffold	6	333.5	109.05x	83.30%	2	GCF_015559305.1
S. constellatus		FDAARGOS_1015	2 038 583	38	1 875	73	113	Complete	1	2038.6	1922.73x	99.99%	3	GCF_016127875.1
S. constellatus		FDAARGOS_1156	1 903 262	38	1 741	74	83	Complete	1	1903.3	2135.84x	100.00%	3	GCF_016725005.1
S. constellatus		FDAARGOS_1208	1 978 680	38	1 827	74	124	Complete	1	1978.7	1928.66x	100.00%	3	GCF_016889885.1
S. constellatus		11-6117	1 869 421	38	1 757	49	106	Contig	3	1900.0	244.4 kb		2	GCF_019336935.1
S. constellatus		S60	1 892 624	38	1 772	47	90	Scaffold	14	1000.0	132x	84.64%	1	GCF_023109175.1
S. constellatus		S54	1 927 926	38	1 827	49	85	Scaffold	17	284.4	102x	84.21%	1	GCF_023109215.1
S. constellatus		S55	1 993 590	38	1 867	48	90	Scaffold	23	408.7	225x	88.08%	2	GCF_023109295.1
S. constellatus		S34	1 818 626	38	1 734	39	88	Scaffold	15	461.6	61x	86.54%	1	GCF_023109615.1
S. constellatus	***constellatus***	**CCUG 24889**	**1 901 581**	**38**	**1 738**	**74**	**84**	**Complete**	**1**	**1901.6**		**99.72%**	**3**	**GCF_023167545.1**
S. constellatus		TCV107	1 980 997	38	1 828	74	101	Complete	1	1981.0	125x	83.58%	3	GCF_024399395.1
S. constellatus		AM109-96	1 821 433	38	1 700	50	69	Scaffold	15	293.1	100x	85.66%	1	GCF_027723325.1
S. constellatus		UMB8371	1 830 535	38	1 704	30	79	Contig	27	246.1	888.45x	85.78%	1	GCF_030215825.1
S. constellatus		SMC7155	1 826 913	38	1 694	48	92	Contig	37	75.4	908x	97.79%	2	GCF_030676655.1
S. constellatus		1033st1_A6_1033SCRN_220408	1 934 643	38	1 822	40	81	Scaffold	26	220.5	29.9x	85.72%	1	GCF_039059945.1
S. constellatus		1033st1_F12_1033SCRN_220408	1 925 965	38	1 816	48	78	Scaffold	24	220.1	30.2x	85.77%	1	GCF_039059995.1
S. constellatus		20925_1_21	1 949 580	38	1 812	39	87	Contig	15	256.4	100x	85.90%	2	GCF_041432565.1
S. constellatus		NCTC11325	1 906 855	38	1 743	74	86	Contig	2	1900.0	100x	100.00%	3	GCF_900459125.1
S. constellatus		SS_Bg39	2 040 680	39	1 937	57	92	Contig	37	108.1	209x	70.52%	1	GCF_902167705.1

Table S4. List and source of gene queries utilized in the study.

Gene Name	Locus Tag	Organism	Accession	Gene Start	Gene Stop
comA	AT689_RS02160	*S. pneumoniae* NCTC7465	NZ_LN831051.1	418894	421047
comB	AT689_RS02155	*S. pneumoniae* NCTC7465	NZ_LN831051.1	417532	418881
comC $_{SP}$	AT689_RS02690	*S. pneumoniae* NCTC7465	NZ_LN831051.1	500327	500452
comD	AT689_RS02695	*S. pneumoniae* NCTC7465	NZ_LN831051.1	500473	501798
comE	AT689_RS02700	*S. pneumoniae* NCTC7465	NZ_LN831051.1	501795	502547
comW $_{SP}$	AT689_RS02540	*S. pneumoniae* NCTC7465	NZ_LN831051.1	474769	475005
comW $_{SA}$	FGK97_RS09015	*S. anginosus* NCTC11064	NZ_LR594037.1	1782424	1782654
comX1	AT689_RS02600	*S. pneumoniae* NCTC7465	NZ_LN831051.1	482441	482920
comX2	AT689_RS03915	*S. pneumoniae* NCTC7465	NZ_LN831051.1	741124	741603
comFA	AT689_RS02850	*S. pneumoniae* NCTC7465	NZ_LN831051.1	528548	529846
comFC	AT689_RS02855	*S. pneumoniae* NCTC7465	NZ_LN831051.1	529843	530505
comEA	AT689_RS09050	*S. pneumoniae* NCTC7465	NZ_LN831051.1	1710010	1710660
comEC	AT689_RS09045	*S. pneumoniae* NCTC7465	NZ_LN831051.1	1707786	1710026
comGA	AT689_RS03685	*S. pneumoniae* NCTC7465	NZ_LN831051.1	701239	702180
comGB	AT689_RS03690	*S. pneumoniae* NCTC7465	NZ_LN831051.1	702128	703144
comGC	AT689_RS03695	*S. pneumoniae* NCTC7465	NZ_LN831051.1	703146	703472
comGD	AT689_RS03700	*S. pneumoniae* NCTC7465	NZ_LN831051.1	703465	703869
comGE	AT689_RS03705	*S. pneumoniae* NCTC7465	NZ_LN831051.1	703832	704134
comGF	AT689_RS03710	*S. pneumoniae* NCTC7465	NZ_LN831051.1	704097	704558
comGG	AT689_RS03715	*S. pneumoniae* NCTC7465	NZ_LN831051.1	704536	704949
cinA	AT689_RS04290	*S. pneumoniae* NCTC7465	NZ_LN831051.1	804272	805528
coiA	AT689_RS08930	*S. pneumoniae* NCTC7465	NZ_LN831051.1	1687997	1688950
cclA	AT689_RS05025	*S. pneumoniae* NCTC7465	NZ_LN831051.1	926954	927613
endA	AT689_RS04210	*S. pneumoniae* NCTC7465	NZ_LN831051.1	784635	785459
radA	AT689_RS02250	*S. pneumoniae* NCTC7465	NZ_LN831051.1	434582	435943
recA	AT689_RS04295	*S. pneumoniae* NCTC7465	NZ_LN831051.1	805583	806749
dprA	AT689_RS07605	*S. pneumoniae* NCTC7465	NZ_LN831051.1	1415325	1416173
ssbB	AT689_RS04440	*S. pneumoniae* NCTC7465	NZ_LN831051.1	826850	827245
qsrA	AT689_RS05575	*S. pneumoniae* NCTC7465	NZ_LN831051.1	1036194	1037087
cbpA	SPD_RS10670	*S. pneumoniae* D39	NC_008533.2	1995045	1997150
cbpB	SPD_RS08840	*S. pneumoniae* D39	NC_008533.2	1670508	1670969
cbpC	SPD_RS01875	*S. pneumoniae* D39	NC_008533.2	349486	350502
cbpD	SPD_RS10730	*S. pneumoniae* D39	NC_008533.2	2006513	2007859
cbpE	SPD_RS04410	*S. pneumoniae* D39	NC_008533.2	838512	840395
cbpF	SPD_RS01940	*S. pneumoniae* D39	NC_008533.2	358923	359807
cbpG	SPD_RS01935	*S. pneumoniae* D39	NC_008533.2	358079	358904
lytA	AT689_RS04305	*S. pneumoniae* NCTC7465	NZ_LN831051.1	808802	809758
lytC	AT689_RS06215	*S. pneumoniae* NCTC7465	NZ_LN831051.1	1159820	1161325
lytF $_{SG}$	SGO_RS10250	*S. gordonii* CH1	NC_009785.1	2155996	2157645
lytF $_{SA}$	HMPREF9966_RS05520	*S. anginosus* SK52	NZ_AFIM01000066.1	27278	29212
comM	AT689_RS04270	*S. pneumoniae* NCTC7465	NZ_LN831051.1	801506	802126
def2	AT689_RS06330	*S. pneumoniae* NCTC7465	NZ_LN831051.1	1183011	1183421
dut	AT689_RS02260	*S. pneumoniae* NCTC7465	NZ_LN831051.1	436474	436917
blpA	AT689_RS11025	*S. pneumoniae* NCTC7465	NZ_LN831051.1	2082276	2084429
blpB	AT689_RS11030	*S. pneumoniae* NCTC7465	NZ_LN831051.1	2084440	2085801
blpC $_{SP}$	AT689_RS11035	*S. pneumoniae* NCTC7465	NZ_LN831051.1	2085858	2086013
blpC $_{SA}$	FGK97_RS02595	*S. anginosus* NCTC11064	NZ_LR594037.1	510497	510646
blpH $_{SP}$	AT689_RS11040	*S. pneumoniae* NCTC7465	NZ_LN831051.1	2086057	2087397
blpH $_{SA}$	FGK97_RS02600	*S. anginosus* NCTC11064	NZ_LR594037.1	510691	511998
blpR	AT689_RS11045	*S. pneumoniae* NCTC7465	NZ_LN831051.1	2087411	2088148
blp3.1		*S. anginosus* BSU 1211	MZ766502.1	286	516
blp3.2		*S. anginosus* BSU 1211	MZ766502.1	746	1066
blp3.3		*S. anginosus* BSU 1211	MZ766502.1	1218	1508
blp3.4		*S. anginosus* BSU 1211	MZ766502.1	1560	1814
blp3.5		*S. anginosus* BSU 1211	MZ766502.1	1801	2187
blp3.6		*S. anginosus* BSU 1211	MZ766502.1	2209	2433
silX		*S. anginosus* BSU 1211	MZ766502.1	2665	3378
silA		*S. anginosus* BSU 1211	MZ766502.1	3541	4293
silB		*S. anginosus* BSU 1211	MZ766502.1	4298	5605
silCR		*S. anginosus* BSU 1211	MZ766502.1	5650	5799
silD		*S. anginosus* BSU 1211	MZ766502.1	5892	7253
silE		*S. anginosus* BSU 1211	MZ766502.1	7264	9414

Footnotes in gene name indicate the same gene target using gene sequences from different species

$_{SP}$: *Streptococcus pneumoniae* . $_{SA}$: *Streptococcus anginosus* . $_{SG}$: *Streptococcus gordonii* .

Table S5. Comparative analysis of exporter similarity.

Species	Subsp.	Strain	*comA* SP	*blpA* SP	*silE* SA	*comB* SP	*blpB* SP	*silD* SA
Streptococcus constellatus		1001254J_160919_C10	70.59%	82.72%	87.56%	42.28%	59.51%	80.92%
Streptococcus constellatus		1033st1_A6_1033SCRN_220408	70.78%	83.75%	89.90%	42.88%	60.07%	81.84%
Streptococcus constellatus		1033st1_F12_1033SCRN_220408	70.78%	83.75%	89.90%	42.88%	60.07%	81.84%
Streptococcus constellatus		11-6117	70.95%	83.92%	90.34%	42.92%	60.29%	82.45%
Streptococcus constellatus		20925_1_21	70.96%	83.93%	90.16%	42.56%	59.92%	82.44%
Streptococcus constellatus		317_SINT	70.39%	83.25%	89.65%	42.59%	60.18%	81.93%
Streptococcus constellatus		783_SANG	70.96%	83.93%	90.16%	42.56%	59.92%	82.44%
Streptococcus constellatus		925_SCON	70.74%	83.65%	90.07%	42.47%	59.86%	81.78%
Streptococcus constellatus		AM109-96	70.63%	83.89%	89.92%	42.70%	60.25%	82.19%
Streptococcus constellatus		D6t1_180914_C10	70.80%	82.93%	87.77%	42.32%	59.51%	80.70%
Streptococcus constellatus		FDAARGOS_1015	70.34%	83.37%	89.43%	42.51%	60.24%	82.44%
Streptococcus constellatus		FDAARGOS_1156	70.83%	83.39%	88.06%	42.62%	60.48%	82.25%
Streptococcus constellatus		FDAARGOS_1208	70.37%	83.41%	89.47%	42.51%	60.24%	82.44%
Streptococcus constellatus		KCOM 1039	-	-	-	42.45%	60.12%	82.18%
Streptococcus constellatus		KCOM 1650	70.95%	83.92%	90.34%	42.53%	60.53%	82.08%
Streptococcus constellatus		NCTC11325	70.83%	83.39%	88.06%	42.57%	60.43%	82.19%
Streptococcus constellatus		S34	70.72%	83.55%	89.89%	41.87%	59.23%	80.81%
Streptococcus constellatus		S54	70.29%	83.34%	89.95%	42.83%	59.99%	82.09%
Streptococcus constellatus		S55	70.36%	83.63%	89.74%	42.57%	60.34%	81.88%
Streptococcus constellatus		S60	70.91%	84.13%	90.36%	42.61%	59.80%	82.19%
Streptococcus constellatus		SMC7155	70.83%	83.39%	88.06%	42.62%	60.48%	82.25%
Streptococcus constellatus		SS_Bg39	71.26%	84.02%	95.68%	42.23%	60.74%	92.72%
Streptococcus constellatus	*constellatus*	13-11-14	70.79%	83.85%	90.16%	42.62%	60.22%	82.21%
Streptococcus constellatus	*constellatus*	ATCC 27823	70.67%	83.23%	91.47%	42.83%	60.24%	82.57%
Streptococcus constellatus	***constellatus***	**CCUG 24889**	**70.07%**	**83.21%**	**89.76%**	**42.63%**	**60.10%**	**82.27%**
Streptococcus constellatus	*constellatus*	SK53	70.83%	83.39%	88.06%	42.62%	60.48%	82.25%
Streptococcus constellatus	*pharyngis*	15-01-28	70.83%	83.39%	88.06%	42.62%	60.48%	82.25%
Streptococcus constellatus	*pharyngis*	C1050	70.83%	83.39%	88.06%	42.62%	60.48%	82.25%
Streptococcus constellatus	*pharyngis*	C232	70.43%	83.46%	89.32%	42.51%	60.24%	82.44%
Streptococcus constellatus	*pharyngis*	C818	70.37%	83.41%	89.47%	42.51%	60.24%	82.44%
Streptococcus constellatus	*pharyngis*	SK1060	70.37%	83.41%	89.47%	40.81%	58.34%	79.42%
Streptococcus constellatus		TCV107	70.37%	83.41%	89.47%	40.81%	58.34%	79.42%
Streptococcus constellatus		UMB8371	70.37%	83.41%	89.47%	42.51%	60.24%	82.44%

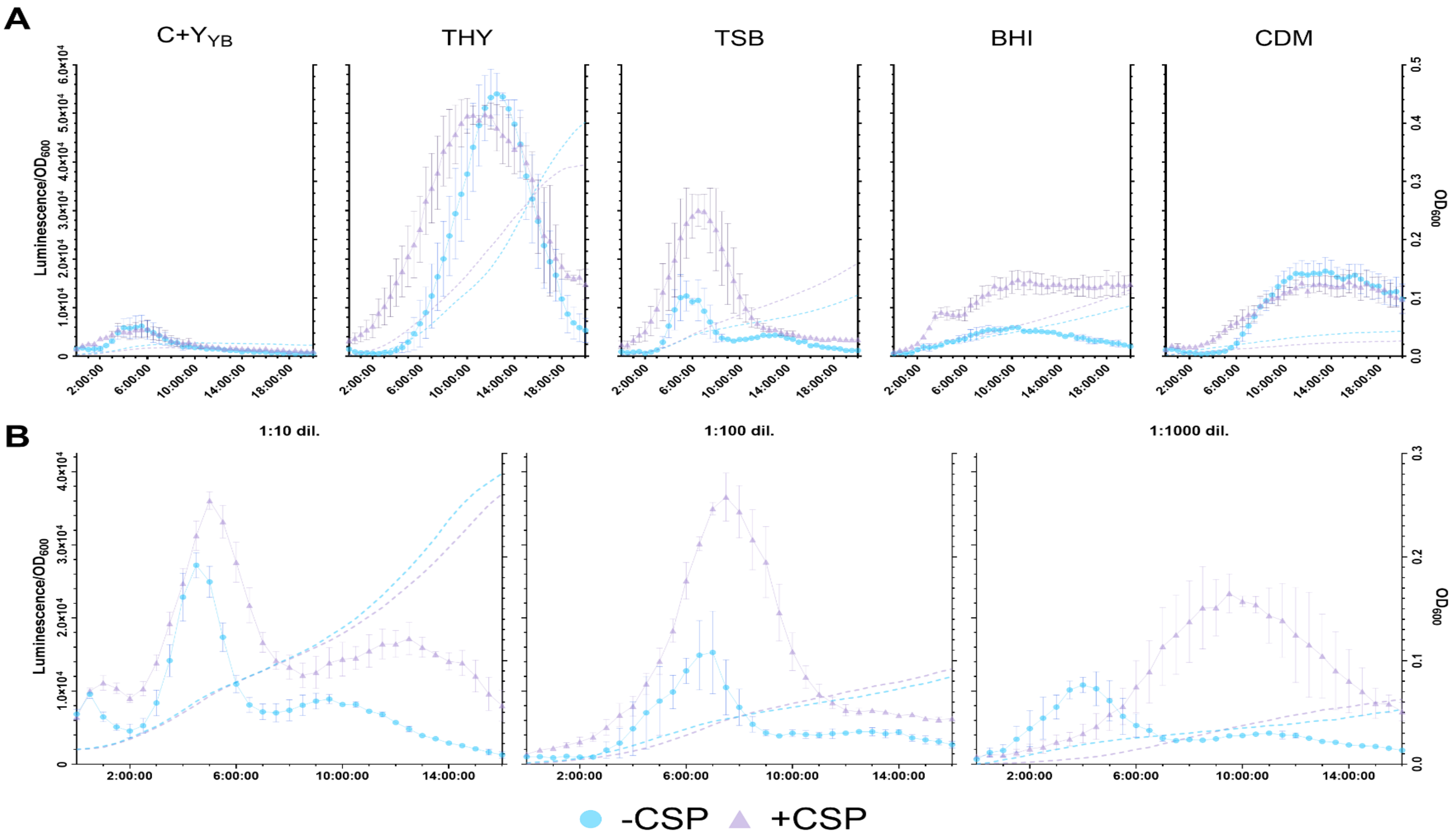

Fig S1. Influence of media and dilution factors on *sigX* expression. **(A)** Comparative *sigX* expression profiles across different growth media ($C{+}Y_{YB}$, THY, TSB, BHI, and CDM) ±CSP. **(B)** Effect of various inoculation dilutions (1:10, 1:100, and 1:1000) in TSB on the timing and intensity of the competence response. All data represent mean ± SEM of three independent biological experiments.
